# X2BR: High-fidelity 3D bone reconstruction from a planar X-ray image with hybrid neural implicit methods

**DOI:** 10.1007/s11517-026-03549-5

**Published:** 2026-03-26

**Authors:** Gokce Guven, Fatih Ugurdag, Hasan F. Ates

**Affiliations:** 1 Dept. of Computer Science, Ozyegin University, Istanbul, 34794 Türkiye; 2 Dept. of Electrical & Eletronics Eng., Ozyegin University, Istanbul, Türkiye; 3 Dept. of Artificial Intel. & Data Eng., Ozyegin University, Istanbul, Türkiye

**Keywords:** Bone reconstruction, X-ray, ConvNeXt, Non-rigid registration, Implicit networks

## Abstract

**Abstract:**

Accurate 3D bone reconstructions are critical for surgical planning, implant design, and tracking musculoskeletal disorders. 3D bone reconstruction from a single planar X-ray remains challenging due to anatomical complexity and limited input. We propose X2BR, a hybrid neural implicit framework combining continuous volumetric reconstruction (X2B) with template-guided non-rigid registration (R). X2B employs a ConvNeXt-based encoder to extract spatial features from X-rays and predict high-fidelity 3D bone occupancy fields without relying on statistical shape models. To refine anatomical accuracy, X2BR integrates a patient-specific template mesh, constructed using YOLOv9-based detection and the SKEL biomechanical skeleton model. The coarse reconstruction is aligned to the template using geodesic-based coherent point drift, enabling anatomically consistent 3D bone volumes. Experimental results show that X2B achieves the highest numerical accuracy, with an IoU of 0.952 and Chamfer-L1 distance of 0.005, outperforming recent baselines including X2V and D2IM-Net. Building on this, X2BR incorporates anatomical priors via YOLOv9-based detection and biomechanical template alignment, leading to reconstructions that offer superior anatomical realism, especially in rib curvature and vertebral alignment, while slightly lower in IoU (0.875). This accuracy vs. visual consistency trade-off between X2B and X2BR highlights the value of hybrid frameworks for clinically relevant 3D reconstructions.

**Graphical Abstract:**

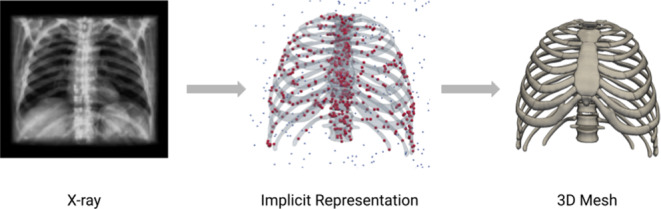

## Introduction

Neural implicit representations have emerged as a powerful paradigm for high-fidelity 3D modeling by representing shapes as continuous functions, enabling precise reconstruction, smooth interpolation, and novel view synthesis without explicit parameterization. Their ability to model fine geometric details has led to widespread applications across computer vision, robotics, and increasingly, medical imaging.

In the medical domain, single-image 3D reconstruction using deep learning has significantly advanced spatial reasoning from sparse 2D data, supporting applications in surgical planning, diagnostic modeling, and patient-specific treatment design. Neural implicit methods, in particular, offer volumetric reconstructions with sub‑voxel accuracy, enabling detailed anatomical analysis. Unlike voxel‑based approaches, they parametrize shape as a continuous field that can be evaluated at arbitrary sampling resolutions at inference time, but they still do not inherently encode anatomical constraints or biomechanical plausibility. Integrating prior knowledge—such as template-based anatomy or landmark-driven registration—is thus essential to ensure clinically meaningful outputs.

Recent advances underscore the versatility of neural implicit methods in healthcare. For instance, X2Teeth reconstructs individual teeth from panoramic radiographs [1], and Oral-3Dv2 employs implicit coordinate fields for full dental arch modeling [2]. ToothInpainter fuses partial 3D models with 2D projections to recover complete dental structures, including roots [3]. Beyond dentistry, MedNeRF synthesizes CT-like views from sparse X-rays [4], ImplicitVol reconstructs ultrasound volumes without voxel grids [5], SAX-NeRF applies line-based transformers for sparse-view radiographs [6], and SNAF enhances cone-beam CT using neural attenuation fields [7]. Collectively, these studies demonstrate the transformative potential of neural implicit modeling in reconstructive medical imaging.

This study introduces X2B and X2BR, two complementary neural implicit frameworks for reconstructing 3D skeletal anatomy from a single planar X-ray. X2B employs a ConvNeXt-based encoder [8] to extract hierarchical spatial features and predict continuous occupancy fields for complex bony structures, such as vertebrae and ribs, without relying on voxel grids or statistical templates. To improve anatomical fidelity, X2BR augments this output via non-rigid registration to a biomechanical skeleton using an accelerated geodesic-based coherent point drift algorithm (GBCPD++) [9]. This hybrid formulation refines reconstructions toward anatomical consistency and accommodates patient-specific variations.

Quantitative evaluations on clinical datasets demonstrate that X2B outperforms existing methods across volumetric IoU, Chamfer-L1 distance, and F-score. While X2BR yields slightly lower numerical accuracy, it substantially improves anatomical plausibility by leveraging biomechanical priors. Together, these frameworks support robust, high-resolution, and anatomically consistent 3D skeletal reconstructions from sparse imaging data—enabling applications in orthopedic assessment, surgical planning, and patient-specific biomechanical simulation.

In summary, the contributions of this study are as follows:

- A hybrid neural implicit framework (X2B + X2BR) is proposed for 3D skeletal reconstruction from a single planar X-ray.
- Continuous occupancy fields and a ConvNeXt-based encoder are employed in X2B for high-fidelity, template-free modeling.
- Anatomical consistency is enhanced in X2BR through non-rigid registration to a biomechanical prior.
- A large-scale clinical dataset of 3D bone meshes paired with DRRs (digitally reconstructed radiograph) is introduced.
- State-of-the-art performance is achieved in terms of IoU, Chamfer-L1, and F-score.

The remainder of this paper is organized as follows. Section 2 reviews related work on single-view 3D reconstruction, X-ray–based bone modeling, and implicit neural representations. Section 3 details the proposed X2B and X2BR frameworks, including network architectures, training, registration, and dataset construction. Section 4 describes the experimental protocol, baseline methods, evaluation metrics, and quantitative and qualitative results. Section 5 concludes with a discussion of clinical implications and directions for future research.

## Related work

### Single-view reconstruction with implicit surface representations

Neural implicit methods commonly use multi layer perceptron to represent occupancy probabilities or signed distance functions (SDFs) for 3D reconstruction from single images [10, 11]. While previous neural implicit approaches effectively reconstruct general 3D shapes, they fail to address the anatomical complexity of skeletal structures from single planar X-rays; our proposed X2B and X2BR frameworks specifically bridge this gap, improving reconstruction accuracy for complex skeletal anatomies.

Building on these foundations, recent convolutional neural network (CNN)-based methods such as DISN [11], MDISN [12], and Ray-ONet [13] improve reconstruction fidelity but continue to face limitations in accurately modeling complex geometries. More advanced frameworks like D2IM-Net [14], ED2IF2-Net [15], G2IFu [16], and LIST [17] further enhance topological accuracy and surface detail reconstruction. However, these approaches are primarily designed for general-purpose 3D reconstruction and remain insufficient when applied to anatomically intricate medical structures from sparse, single-view X-ray data.

### X-ray to 3D bone reconstruction with deformation learning

Statistical Shape Models (SSMs) utilize atlases derived from healthy samples to model mean anatomical shapes and variations [18, 19]. Aubert et al. [20] combined SSMs with CNN-based landmark detection for automated 3D spinal reconstruction. Jiang et al. [21] proposed a 2D/3D registration method for spinal geometry reconstruction from frontal X-rays, while X23D [22] integrated multi-view stereo and X-ray calibration for intraoperative vertebrae modeling.

More recent deep learning approaches also leverage deformation modeling. BX2S-Net [23] employs bi-planar X-rays with encoder-decoder architectures and attention mechanisms for improved semantic alignment. Similarly, Yang et al. [24] adapted X2CT-GAN [25] to reconstruct spinal structures from bi-planar radiographs. For single-view reconstruction, the approach in [26] utilizes deep learning with deformation parameters to reconstruct accurate 3D femoral models, while FracReconNet [27] improves fracture reconstruction accuracy by augmenting training data.

Existing methods often depend on bi-planar inputs, explicit deformation models, or statistical shape templates, limiting their generalizability and patient specificity. In contrast, X2B reconstructs high-fidelity 3D skeletal structures directly from single planar X-rays without requiring such priors. X2BR further enhances anatomical consistency by integrating a patient-specific template and geodesic-based non-rigid alignment. Together, they combine the strengths of data-driven reconstruction and anatomy-aware refinement for robust single-view bone modeling.

### Implicit neural representations in medical imaging

Recent developments in implicit neural representations have substantially advanced medical image reconstruction. X2Teeth [1] integrates feature extraction, segmentation, and reconstruction networks for dental modeling. Oral-3Dv2 [2] maps 2D coordinates to 3D voxel densities using dynamic sampling, while ToothInpaintor [3] reconstructs complete dental anatomies, including roots. MedNeRF [4] synthesizes CT-like projections from X-rays, and ImplicitVol [5] reconstructs 3D ultrasound volumes without voxel grids. SAX-NeRF [6] and SNAF [7] further extend implicit modeling to sparse-view X-rays through specialized sampling and field augmentation. Our prior work, X2V [28], employs a ViT-based occupancy network to reconstruct 3D lung volumes from a single X-ray, achieving state-of-the-art accuracy without relying on mesh templates.

However, most existing methods are limited to soft tissues, isolated anatomical regions, or assume regular and high-contrast structures. For instance, X2V is restricted to air-filled organs like the lungs. In contrast, the proposed X2BR framework extends implicit modeling to complex skeletal anatomy, including articulated structures such as vertebrae, by integrating biomechanical templates and non-rigid registration. This enables anatomically consistent 3D reconstruction from a single planar X-ray, even without multi-view inputs or statistical shape priors.

Conventional 3D reconstruction methods based on statistical shape models, atlas-based registration, or voxelized CNNs typically require multi-view imaging and strong priors, which limit patient-specific accuracy from a single X-ray. In contrast, the proposed X2B and X2BR employ continuous neural implicit fields with anatomy-aware registration to achieve high-fidelity skeletal reconstruction from single-view radiographs.

**Fig. 1:**
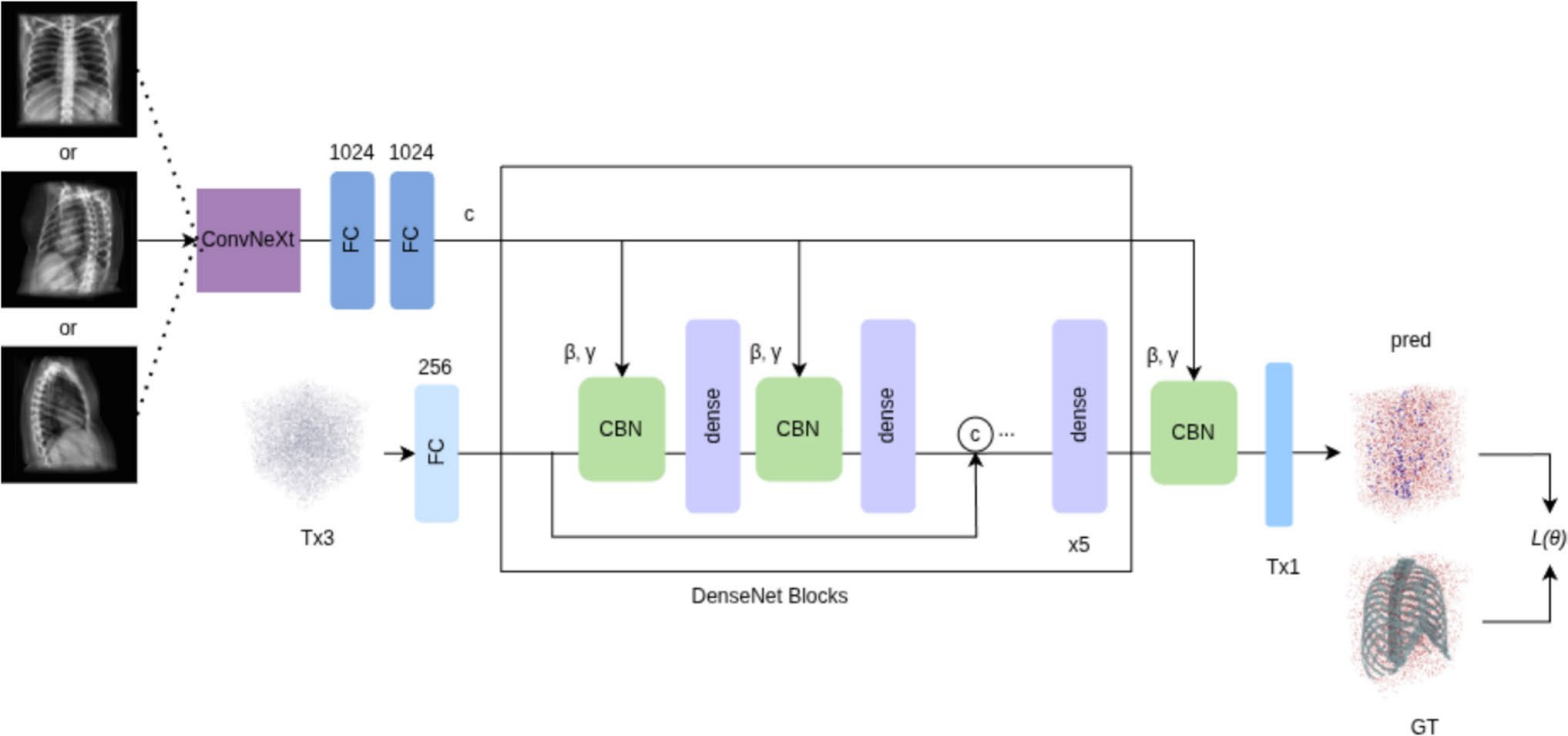
X2B network training pipeline. The network takes a DRR as input and uses a ConvNeXt backbone and FC (fully-connected) layers for feature extraction. The extracted features are passed through dense blocks equipped with Conditional Batch Normalization (CBN) to refine latent representations; the CBN scale/shift parameters $(\gamma ,\beta )$ are generated from the conditioning features and applied within the normalization layers. Here, ***T*** denotes the number of sampled 3D query points. $T \times 3$ refers to dimensions of the query-point tensor $P \in \mathbb {R}^{T \times 3}$ containing (*x*, *y*, *z*) coordinates. Numeric labels (e.g., 256/1024) indicate feature dimensionality/channels, whereas blocks denote processing units

## Proposed method

Accurate 3D bone reconstruction from single planar X-rays remains challenging due to anatomical complexity and overlapping intensities. This study proposes X2B and X2BR, two neural implicit models enabling precise, patient-specific reconstructions.

### X2B network architecture

X2B predicts occupancy probabilities for *T* sampled 3D points from a $224 \times 224$ DRR input, indicating whether each point lies inside the bone surface (see Fig. 1). We use a ConvNeXt encoder [8], a modern ResNet-style CNN backbone, to extract hierarchical features from the DRR; this provides richer context than a conventional CNN and helps resolve thin, low-contrast rib and vertebral structures. This is mainly due to ConvNeXt’s stronger multi-scale feature aggregation and larger effective receptive fields in its stage-wise design, which is beneficial under projection superposition in DRRs. The encoder compresses the image into a 1024-dimensional latent vector, which is concatenated with 3D query points and passed through DenseNet blocks with Conditional Batch Normalization (CBN). The resulting occupancy field [10] implicitly defines the bone surface, and DRRs from multiple projection angles are used during training to encourage view-consistent 3D reconstruction.

**Fig. 2:**
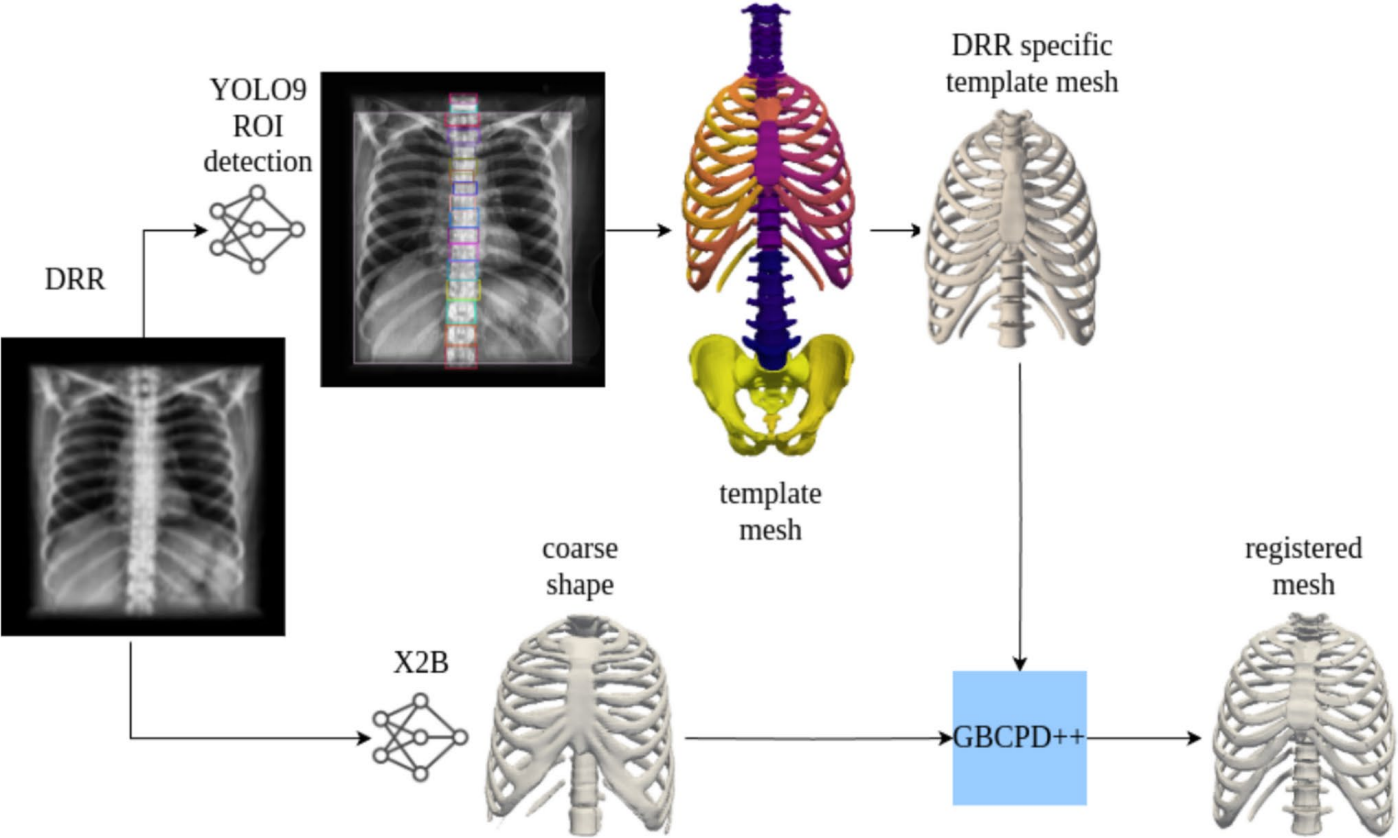
X2BR model architecture. Anterior-posterior DRR is used as input for both YOLOv9 and X2B for inference. The regions are detected via YOLOv9 network and DRR specific template mesh is extracted from the template mesh model using the detected regions. DRR specific template model is registered to the coarse shape, which is the output of the X2BR model

### X2BR network architecture

X2BR refines X2B outputs via non-rigid registration (Fig. 2). A fine-tuned YOLOv9 model detects vertebrae (C1–L5) and rib-pairs in DRRs, using ground-truth 2D bounding boxes generated by forward-projecting segmented CT meshes (Section 3.6.1). YOLOv9 achieves $\textrm{mAP}_{50\text {--}95}=0.964$ on held-out patients after 300 epochs. Detected regions are mapped to subject-specific template meshes derived from the SKEL Biomechanical Skeleton Model (BSM) [29], built in OpenSim [30]. Coarse predictions from X2B are aligned to these templates using the GBCPD++ algorithm [9], addressing complex deformations.

The combination of ConvNeXt encoding, CBN normalization, YOLOv9 object detection, and GBCPD++ alignment enables X2B and X2BR to address the limitations of traditional methods. These models deliver precise 3D reconstructions, making them suitable for applications in surgical planning, biomechanical analysis, and personalized treatment.

**Fig. 3:**
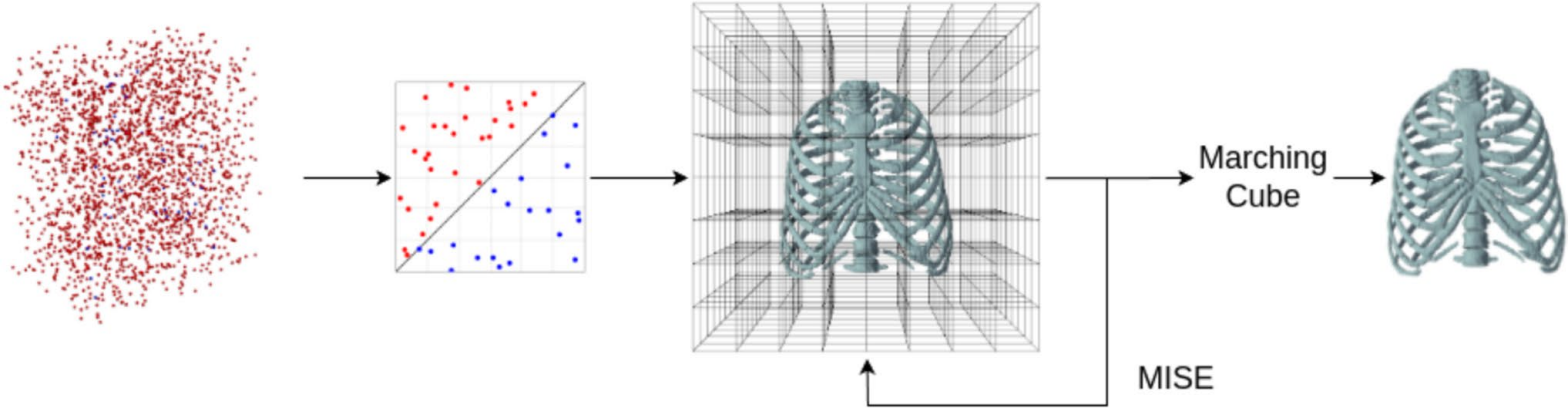
For 3D mesh inference with *X2B* model, a modified Multiresolution IsoSurface Extraction (MISE) algorithm for high-resolution mesh extraction is integrated, starting with a base resolution and evaluating against the occupancy network. The occupancy threshold is set at $\tau = 0.2$ for balance in accuracy and completeness. The process involves subdividing voxels until the desired resolution is reached, using Marching Cubes for mesh generation, and refining the mesh with Fast-Quadric-Mesh-Simplification and gradient optimization. Our method achieves efficient and accurate mesh inference, optimized for an initial resolution of $32^3$, and is capable of extracting mesh normals effectively

### Training of X2B

The X2B network is trained to approximate the binary occupancy function $o: \mathbb {R}^3 \rightarrow \{0,1\}$ associated with each DRR. For a given DRR $x_i$ (captured at a random angle), we uniformly sample *T* 3D points $\{p_{ij}\}_{j=1}^T$ inside the normalized bounding box and assign binary occupancy labels $o_{ij} = o(p_{ij})$ from the ground-truth mesh. In our implementation, all sampled points for a given DRR share the same conditioning image $x_i$ and its encoding $c_i = \textrm{Enc}(x_i)$; thus the pairs $(p_{ij}, x_i)$ differ only in their 3D location $p_{ij}$.

Following Occupancy Networks [10], we introduce a global latent shape code $z_i \in \mathbb {R}^d$ with a standard normal prior $p_0(z) = \mathcal {N}(0,I)$. A variational encoder $q_\phi (z_i \mid p_i, o_i, c_i)$ infers a Gaussian approximate posterior over $z_i$. Given $z_i$ and the image encoding $c_i$, the decoder $f_\theta$ predicts a logit $\ell _{ij} = f_\theta (p_{ij}, z_i, c_i)$ for each query point, and the corresponding occupancy probability is

1 $$\begin{aligned} \hat{o}_{ij} = \sigma (\ell _{ij}), \end{aligned}$$

where $\sigma (\cdot )$ denotes the sigmoid function.

The reconstruction term is the binary cross-entropy between predicted occupancies and ground-truth labels,

2 $$\begin{aligned} \ell _{\textrm{BCE}}(\ell _{ij}, o_{ij}) = -\, o_{ij} \log \sigma (\ell _{ij}) - (1 - o_{ij}) \log \big (1 - \sigma (\ell _{ij})\big ), \end{aligned}$$

which in practice is computed in a numerically stable logit-based form of the binary cross-entropy loss. Averaging over points and samples in a mini-batch *B*, the total loss is:

3 $$\begin{aligned} L_B(\theta , \phi ) = \frac{1}{|B|} \sum _{i \in B} \left[ \textrm{KL}\big (q_\phi (z_i \mid p_i, o_i, c_i) \,\Vert \, p_0(z)\big ) + \frac{1}{T} \sum _{j=1}^T \ell _{\textrm{BCE}}(\ell _{ij}, o_{ij}) \right] , \end{aligned}$$

which corresponds to the negative evidence lower bound (ELBO) on the conditional occupancy likelihood optimized during training.

For all experiments, the Adam optimizer [8] is employed with a learning rate $\eta = 10^{-4}$ and default PyTorch hyperparameters $\beta _1 = 0.9$, $\beta _2 = 0.999$, and $\epsilon = 10^{-8}$.

### Inference

As illustrated in Fig. 3, 3D meshes are inferred from X2B occupancy predictions using a modified Multiresolution IsoSurface Extraction (MISE) algorithm [10]. The method begins with coarse volumetric discretization, where occupancy values are thresholded ($\tau = 0.2$) to classify voxels as inside or outside, following ONet guidelines [10].

Active voxels are recursively subdivided to reach the target resolution. The Marching Cubes algorithm extracts the surface mesh, which is subsequently refined using Fast-Quadric-Mesh-Simplification via edge contraction and quadric error minimization, followed by gradient-based optimization for improved mesh quality.

### Registration

To refine the coarse implicit reconstruction produced by X2B, X2BR performs non-rigid registration between the DRR-specific biomechanical template mesh and the X2B mesh using the Geodesic-Based Bayesian Coherent Point Drift (GBCPD++) algorithm [9]. Classical Coherent Point Drift (CPD) enforces motion coherence in Euclidean space and, for articulated anatomy, may cause neighboring structures (e.g., adjacent ribs) to deform jointly in anatomically implausible ways. GBCPD++ instead defines motion coherence through geodesic distances on the template surface, promoting deformations that are smooth within each bone while allowing relative motion between distinct anatomical parts.

In our implementation, the vertices of the DRR-specific template mesh serve as the source point set, and the X2B reconstruction is treated as the target point set. GBCPD++ fits a Gaussian mixture model on the source points with a covariance kernel parameterized by geodesic distances and estimates a dense displacement field by maximizing the posterior likelihood of aligning the source to the target. The resulting deformation field is applied to the template mesh to obtain the final registered mesh used in X2BR.

By leveraging geodesic kernels and the scalable optimization strategy of GBCPD++, the registration step corrects residual discrepancies in the coarse X2B output and yields anatomically consistent vertebral and rib alignments, while preserving local geometric detail required for downstream surgical planning and biomechanical analysis.

### Dataset

A custom dataset was curated for this study due to the absence of public paired X-ray and 3D bone mesh datasets. High-resolution CT scans, digitally reconstructed radiographs (DRRs), and watertight bone meshes were systematically processed using a combination of automatic segmentation, rendering, and preprocessing steps to support training and evaluation of the proposed models. The resulting pipeline, detailed in Sections 3.6.1–3.6.3, spans CT acquisition, bone segmentation and mesh generation, occupancy-value computation, DRR synthesis, and DRR annotation for YOLOv9 training.

#### Segmentation with totalsegmentator

The X2B framework focuses on high-contrast skeletal structures such as ribs, costal cartilages, and vertebrae. We employ TotalSegmentator [31], a deep learning–based multi-organ segmentation framework that provides robust 3D delineation of 104 anatomical structures in CT, and use only the thoracic bone labels. Scapulae are excluded due to frequent segmentation errors in the lateral regions.

CT scans are obtained from the National Lung Screening Trial (NLST) subset of The Cancer Imaging Archive (TCIA) [32], a large-scale public repository of low-dose chest CT examinations acquired for lung cancer screening. The dataset preparation pipeline comprises the following steps:

- Collection of thoracic CT scans from the NLST–TCIA cohort.
- Automatic segmentation of thoracic bones and conversion into watertight meshes using TotalSegmentator [31].
- Occupancy value computation from mesh samples within a normalized bounding volume.
- Generation of multi-view DRR images via GPU-accelerated ray casting [33].
- Post-processing of DRRs using Contrast Limited Adaptive Histogram Equalization (CLAHE) to enhance local contrast and highlight bony structures.

In total, 3,640 CT volumes from 964 subjects are processed. Scans with incomplete thoracic coverage or severe artifacts are identified and excluded after visual inspection. We use the off-the-shelf TotalSegmentator model without additional manual 3D annotations; its mean Dice score of 0.943 ± 0.04 for bone structures is taken from the original benchmark reported in [31]. All geometric processing steps—including isotropic resampling, mesh extraction, centering and scaling, occupancy computation, and DRR synthesis—are executed by fully automated scripts, while manual intervention is limited to the initial quality control of CTs (exclusion of corrupted or incomplete scans).

For learning-based experiments, the dataset is partitioned into disjoint training, validation, and test sets at the *patient* level: all CT volumes, meshes, and DRRs belonging to a given subject are assigned to a single split. This preprocessing pipeline produces (i) occupancy labels for X2B, (ii) DRR inputs for all models, and (iii) forward-projected masks used to derive bounding boxes for YOLOv9 training, thereby defining the complete data flow before network training.

#### Bone localization and detection

Anterior–posterior (AP) DRR images at $512 \times 512$ resolution are annotated using the Roboflow platform [34]. A subset of images is first labeled manually to establish high-quality bounding boxes for vertebrae and rib pairs, after which Roboflow’s autolabeler is used to propagate annotations to the remaining images. All automatically generated labels are subsequently reviewed and, when necessary, corrected by an expert annotator. The resulting dataset of curated bounding boxes is then used to train the YOLOv9 detector, yielding robust and accurate bone localization.

#### Occupancy value calculation

Watertightness of the extracted meshes is enforced using the trimesh Python library [10]. All meshes are centered and normalized into a unit bounding box. Following Occupancy Networks (ONet) [10], $32^3$ voxel grids are generated, voxels intersecting the mesh surface are labeled as occupied, and 100,000 random points are sampled and tested for inclusion via ray–mesh intersection counting. During training of X2B, a random subset of 2,048 points per sample is selected from this pool to compute occupancy probabilities. This occupancy computation and subsampling procedure is fully automated and provides the supervision signal required for accurate 3D reconstruction [35].

## Results and discussion

This section describes the baseline methods and evaluation protocol used to assess the proposed X2B and X2BR networks. The 3D reconstruction performance of X2B and X2BR is compared against three recent single-image 2D/3D reconstruction models from the literature: X2V [28], D2IM-Net [14], and ED2IF2-Net [15]. Section 4.3 presents the simulation results and provides a detailed quantitative and qualitative analysis of the findings.

### Comparative studies

This subsection details the X2V, D2IM-Net, and ED2IF2-Net models used for performance comparison. For clarity, we summarize their main characteristics (input type, encoder, and representation) in Table 1. For a fair comparison, all baseline methods (D2IM-Net, ED2IF2-Net, and X2V) are reimplemented and trained on the proposed DRR dataset using the same patient-level training, validation, and test splits as X2B and X2BR.

#### X2V

X2V [28] reconstructs 3D organ volumes from a single X-ray using an implicit occupancy representation, eliminating the need for templates. Unlike D2IM-Net and ED2IF2-Net, it leverages a Vision Transformer (ViT) encoder for enhanced feature extraction and achieves strong performance for X-ray to 3D lung volume reconstruction.

#### D2IM-net

D2IM-Net [14] is a single-view 3D reconstruction network that combines a coarse implicit field with displacement maps for front and back surfaces to recover topological structures and fine details. Using two decoders to extract global and local features, it attains high reconstruction quality in terms of Chamfer Distance (CD), Intersection over Union (IoU), and Edge Chamfer Distance (ECD). Its Laplacian loss improves surface detail representation [11], outperforming DISN.

#### ED2IF2-net

ED2IF2-Net [15] leverages a Pyramid Vision Transformer (PVT) for high-fidelity 3D reconstruction from single RGB images. It disentangles the implicit field into a deformed implicit field for topology and a displacement field for surface details. The architecture comprises three decoders: a coarse shape decoder, a deformation decoder using implicit field deformation blocks (IFDBs), and a surface detail decoder with hybrid attention modules (HAMs). A comprehensive loss function jointly supervises coarse shape and fine details.

**Table 1:** Summary of baseline methods and proposed models

Method	Input type	Encoder	Representation
D2IM-Net [14]	Single RGB / DRR	CNN-based	Coarse implicit field + displacement maps
ED2IF2-Net [15]	Single RGB / DRR	PVT	Deformed implicit field + displacement field
X2V [28]	Single DRR	ViT	Implicit occupancy field
X2B (ours)	Single DRR	ConvNeXt	Implicit occupancy field
X2BR (ours)	Single DRR	ConvNeXt	Implicit occupancy + template-based registration

### Evaluation metrics

The proposed method and baselines are evaluated using the following metrics, following the protocol established in X2V [28]: voxelized Intersection over Union (IoU), Chamfer-L$_1$ (C-L$_1$) distance, F-score, normal consistency (NC), and mean absolute axis errors (maze, maxe, maye).

#### Intersection over Union (IoU)

To compute IoU, both predicted and ground-truth meshes are voxelized with a voxel size of 0.5. Let $V_{\text {pred}}$ and $V_{\text {gt}}$ denote the sets of occupied voxels for the predicted and ground-truth volumes, respectively. IoU is defined as

4 $$\begin{aligned} \textrm{IoU} = \frac{|V_{\text {pred}} \cap V_{\text {gt}}|}{|V_{\text {pred}} \cup V_{\text {gt}}|}. \end{aligned}$$

Higher IoU values indicate better volumetric overlap.

#### Chamfer-L_1_ distance (C-L_1_)

Chamfer-L$_1$ measures the average bidirectional nearest-neighbor distance between predicted and ground-truth surfaces. Let $\mathcal {P}$ and $\mathcal {G}$ denote point sets sampled from the predicted and ground-truth meshes, respectively. The C-L$_1$ distance is computed as

5 $$\begin{aligned} \mathrm {C\text {-}L}_1(\mathcal {P}, \mathcal {G}) = \frac{1}{|\mathcal {P}|} \sum _{p \in \mathcal {P}} \min _{g \in \mathcal {G}} \Vert p - g\Vert _1 + \frac{1}{|\mathcal {G}|} \sum _{g \in \mathcal {G}} \min _{p \in \mathcal {P}} \Vert g - p\Vert _1. \end{aligned}$$

Lower values correspond to more accurate geometric reconstruction.

#### F-score

Following [28], F-score is computed at a distance threshold $t = 0.02$. Precision and recall are defined as

6 $$\begin{aligned} \textrm{Precision}(t) = \frac{\big |\{p \in \mathcal {P} \mid d(p,\mathcal {G})< t\}\big |}{|\mathcal {P}|}, \\ \textrm{Recall}(t) = \frac{\big |\{g \in \mathcal {G} \mid d(g,\mathcal {P}) < t\}\big |}{|\mathcal {G}|}, \end{aligned}$$

where $d(\cdot ,\cdot )$ denotes the nearest-neighbor distance. The F-score is then

7 $$\begin{aligned} \mathrm {F\text {-}score}(t) = \frac{2\,\textrm{Precision}(t)\,\textrm{Recall}(t)}{\textrm{Precision}(t)+\textrm{Recall}(t)}. \end{aligned}$$

#### Normal Consistency (NC)

NC evaluates the alignment between predicted and ground-truth surface normals. For each point $p \in \mathcal {P}$ with normal $n_{\text {pred}}(p)$, we find its nearest neighbor $g \in \mathcal {G}$ with normal $n_{\text {gt}}(g)$. NC is defined as

8 $$\begin{aligned} \textrm{NC} = \frac{1}{|\mathcal {P}|} \sum _{p \in \mathcal {P}} \big |\, n_{\text {pred}}(p) \cdot n_{\text {gt}}(g(p)) \,\big |, \end{aligned}$$

where *g*(*p*) denotes the nearest ground-truth neighbor of *p*. Values close to 1 indicate well-aligned surface orientations.

#### Mean absolute axis errors (maze, maxe, maye)

Following [19], we also report mean absolute axis errors after rigid alignment of the predicted mesh to the ground truth using ICP. Let $(x^{\text {pred}}_k, y^{\text {pred}}_k, z^{\text {pred}}_k)$ and $(x^{\text {gt}}_k, y^{\text {gt}}_k, z^{\text {gt}}_k)$ denote the coordinates of corresponding vertices. The mean absolute *z*-axis error (maze) is:

9 $$\begin{aligned} \textrm{maze} = \frac{1}{N} \sum _{k=1}^{N} \big |z^{\text {pred}}_k - z^{\text {gt}}_k\big |, \end{aligned}$$

with $\textrm{maxe}$ and $\textrm{maye}$ defined analogously along the *x*- and *y*-axes, respectively. Lower values indicate better spatial alignment along each anatomical axis.

### Reconstruction results

This subsection presents benchmark reconstruction experiments on the held‑out test set, showing that X2B attains the highest IoU and lowest Chamfer distances, yielding anatomically faithful 3D reconstructions. Unless otherwise stated, all quantitative results in this section are reported on a held-out test set that is disjoint from the training and validation subjects, and are obtained from our own implementations of all compared methods trained on the same patient-level split; metrics are given as mean ± standard deviation across test cases.

#### X2B performance analysis

**Fig. 4:**
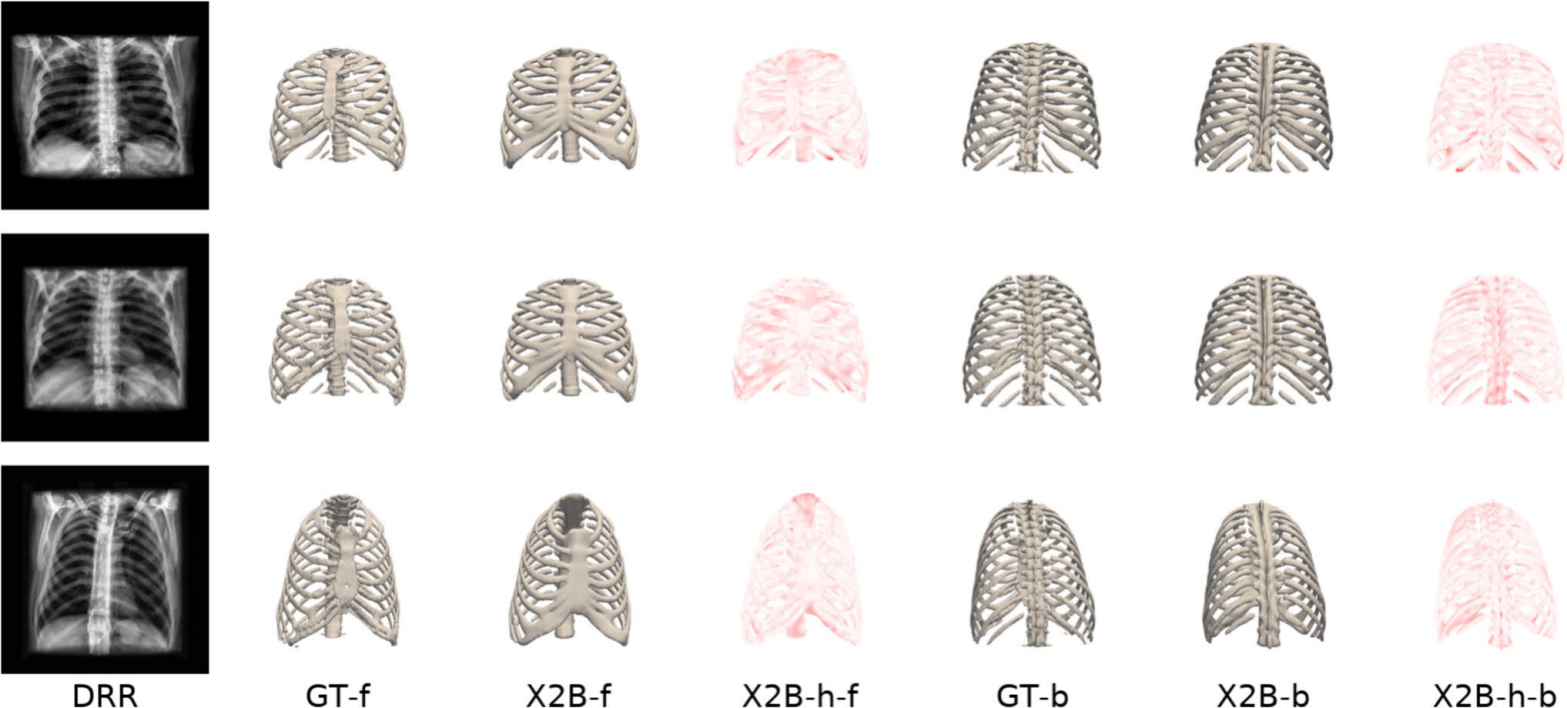
Comparison of X2B and GT. The first column displays the DRR inputs, while the second column presents the GT meshes. The subsequent columns show the X2B reconstructions (X2B-f), X2B heatmaps (X2B-h-f), GT-b, X2B-b, and X2B heatmaps in the back view (X2B-h-b)

Figure 4 illustrates X2B reconstructions compared to GT meshes, showing DRR inputs, front and back views of GT and reconstructed meshes, and heatmaps highlighting reconstruction errors. The mean error is 2.192 mm, with a standard deviation of 1.886 mm, reflecting the model’s alignment accuracy and variability. Positive errors in X2B heatmaps result from over-smoothing in occupancy networks, limited training data resolution, and interpolation effects, which bias reconstructions toward overestimated boundaries.

#### X2B & X2BR comparison with existing methods

**Fig. 5:**
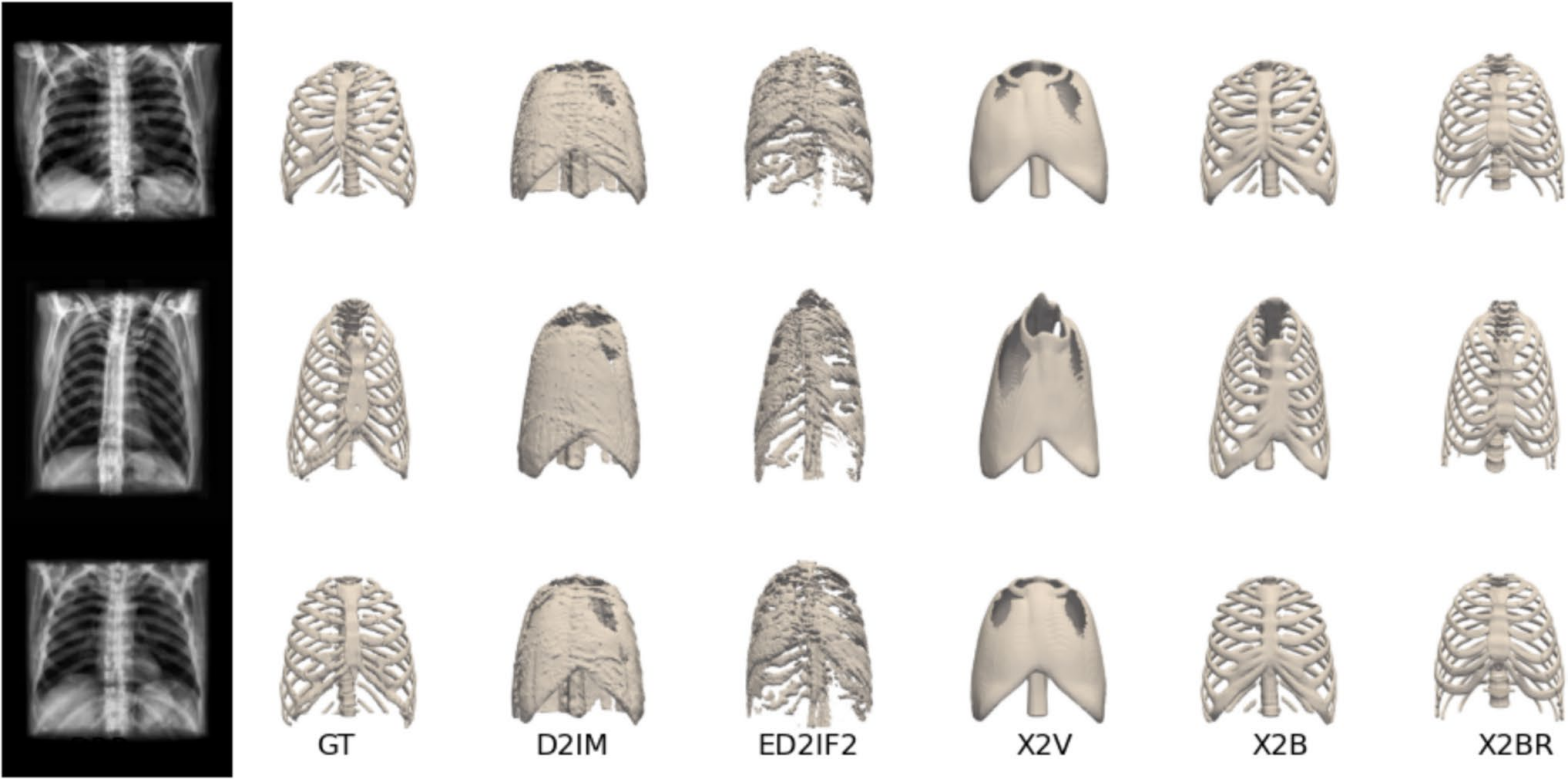
Comparison of reconstruction results across different methods. The figure presents DRR images (leftmost column) and their corresponding GT 3D meshes alongside reconstructed outputs from various methods: D2IM, ED2IF2, X2V, X2B, and X2BR. Each row corresponds to a different DRR input, while the columns illustrate the progression of reconstruction quality across the methods

Figure 5 compares 3D reconstruction results from D2IM-Net, ED2IF2-Net, X2V [28], X2B, and X2BR, all trained on the same dataset using DRRs as inputs for a fair evaluation. X2B and X2BR demonstrate superior performance, with X2BR excelling in preserving fine anatomical features, such as rib curvature and spacing, making it particularly suitable for biomedical applications. In contrast, X2V can reconstruct overall organ topology but lacks fine structural details due to the limitations of its implicit representation. D2IM and ED2IF2 struggle with depth variations, overlapping structures, and complex backgrounds, leading to lower reconstruction accuracy.

Table 2 evaluates reconstruction accuracy based on IoU, Chamfer-L1 distance, F-score, and NC. X2B outperforms all methods, achieving the highest IoU, lowest Chamfer-L1 distance, and highest F-score. X2BR performs competitively, with improved NC.

**Table 2:** Comparison of the methods in terms of various metrics for mesh reconstruction

Method	*IoU*	*Chamfer-L1*	*F-score*	*NC*
*D2IM*	0.903 ± 0.028	0.009 ± 0.001	0.912 ± 0.027	0.499 ± 0.006
*ED2IF2*	0.573 ± 0.041	0.019 ± 0.002	0.567 ± 0.048	0.495 ± 0.003
*X2V*	0.859 ± 0.057	0.009 ± 0.001	0.904 ± 0.050	0.496 ± 0.005
*X2B*	**0.952 ± 0.038**	**0.005 ± 0.001**	**0.974 ± 0.038**	**0.505 ± 0.003**
*X2BR*	0.875 ± 0.036	0.009 ± 0.001	0.913 ± 0.039	0.504 ± 0.003

**Table 3:** Comparison of the methods in terms of maze, maxe, and maye metrics in mm

Method	*maxe*	*maye*	*maze*
*D2IM*	3.468 ± 3.157	2.772 ± 2.474	3.304 ± 3.095
*ED2IF2*	11.871 ± 10.267	6.254 ± 6.655	7.114 ± 6.794
*X2V*	4.545 ± 5.996	4.706 ± 9.352	4.442 ± 6.698
*X2B*	**2.821 ± 2.717**	**2.515 ± 2.320**	**2.590 ± 2.443**
*X2BR*	3.562 ± 3.909	3.667 ± 4.203	3.003 ± 2.793

The reported differences are descriptive; no additional hypothesis testing across multiple training runs was performed, and the reported variation reflects inter-subject variability in the test set. Table 3 compares D2IM, ED2IF2, X2V [28], X2B, and X2BR using maxe, maye, and maze metrics. While X2B achieves the best numerical scores, X2BR provides the most visually faithful reconstructions, especially for rib curvature, spacing, and vertebral alignment (Fig. 5). Its slightly higher errors mainly stem from localized non-rigid deformations that improve anatomical realism but introduce small global misalignments with respect to the ground-truth mesh.

This trade-off reveals a crucial limitation of purely numerical metrics, which can underestimate perceptual or anatomical quality in cases involving fine-grained, patient-specific variations. The template-guided refinement in X2BR enables it to better model true anatomical structure, even if global alignment metrics slightly worsen. Without this qualitative improvement, X2BR’s contribution would be redundant; however, visual comparisons clearly demonstrate its added value in clinical interpretability and anatomical plausibility, which are critical for real-world applications such as surgical planning and biomechanical simulations. Thus, X2B should be preferred when global quantitative accuracy is the primary objective, whereas X2BR is more appropriate when local anatomical fidelity of ribs and vertebrae is critical.

#### Comparison of registration techniques

**Fig. 6:**
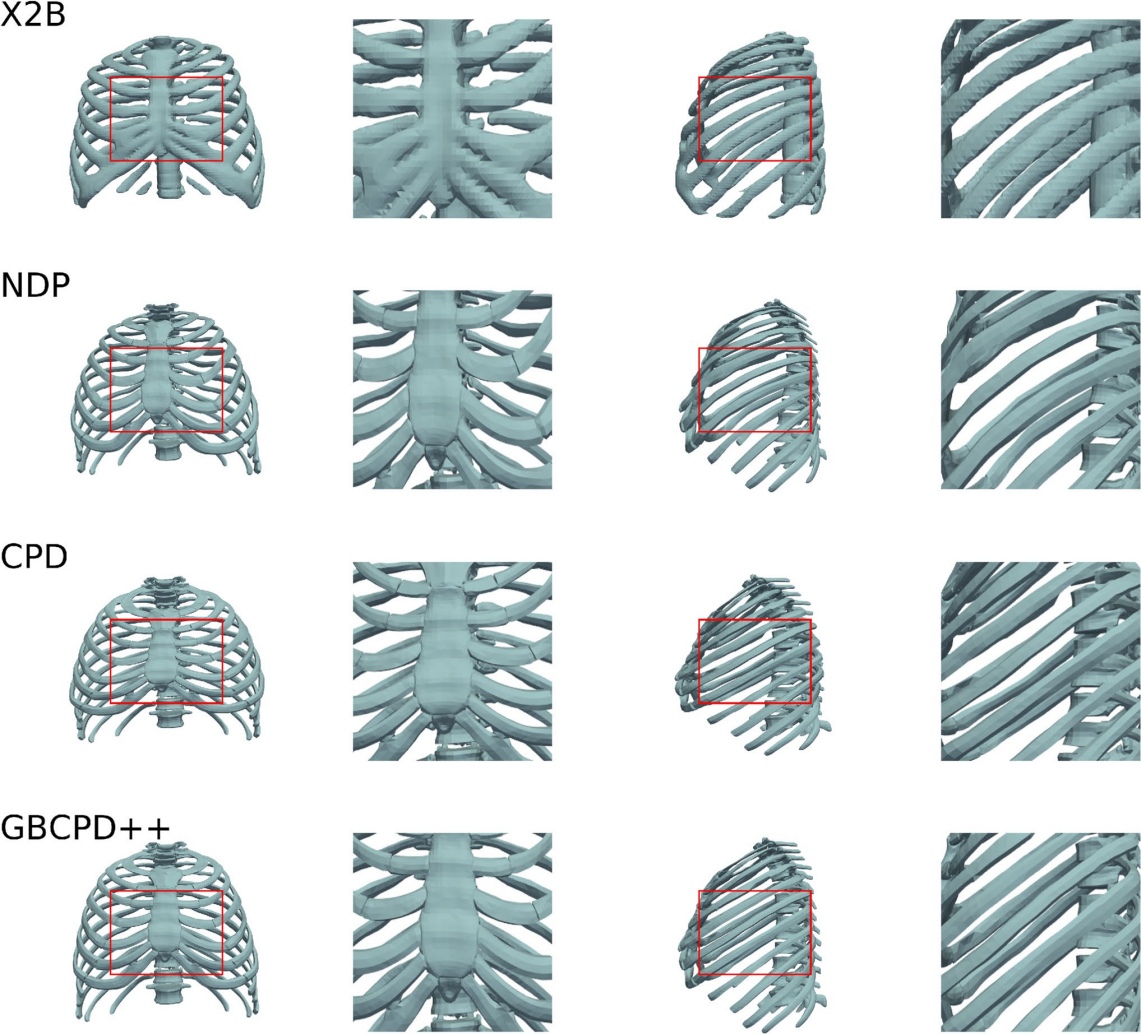
Thorax reconstructions using X2B, NDP, CPD, and GBCPD++. Each row shows full views (front and side) and corresponding zoom-ins highlighting fine anatomical details

Table 4 and Fig. 6 evaluate the effectiveness of different non-rigid registration techniques in aligning reconstructed thorax models. The objective is to assess how well each method preserves anatomical structures while handling global and local deformations.

**Table 4:** Comparison of GBCPD++, NDP, and CPD in terms of various metrics

Method	*IoU*	*Chamfer-L1*	*F-score*	*NC*
*NDP*	0.664 ± 0.111	0.008 ± 0.001	0.637 ± 0.124	0.498 ± 0.003
*CPD*	0.744 ± 0.049	0.014 ± 0.001	0.748 ± 0.051	0.498 ± 0.003
*GBCPD*	0.900 ± 0.019	0.010 ± 0.001	0.931 ± 0.019	0.500 ± 0.004

Table 4 reports only registration methods (NDP, CPD, GBCPD++) applied on the same X2B coarse mesh; the baseline X2B performance without registration is given in Tables 2 and 3. GBCPD++ achieves the best metrics, including IoU, Chamfer-L1 distance, and F-score, demonstrating superior alignment and structural preservation. CPD shows moderate performance, while NDP exhibits significant misalignments, particularly in rib spacing and structural integrity. These results highlight GBCPD++ as the most effective method for accurate non-rigid registration.

#### Single vs. Double DRRs as model input

This subsection reports an ablation variant, X2B2, which extends X2B to bi‑planar input in order to quantify the benefit of adding a second view. X2B2 takes as input two orthogonal DRRs—an anterior–posterior and a lateral projection and fuses them using a multi‑head cross‑attention mechanism for enhanced spatial feature integration.

Tables 5 and 6 compare the performance of X2B and X2B2 in 3D reconstruction tasks. Both models perform exceptionally well, but X2B2 demonstrates slight numerical advantages, achieving higher IoU and lower Chamfer-L1 distance , indicating improved geometric accuracy. X2B2 also attains higher F-score, showcasing its robustness in capturing fine structural details, while both models achieve identical NC scores.

**Table 5:** Comparison of X2B and X2B2 results in terms of various metrics

Method	*IoU*	*Chamfer-L1*	*F-score(t=0.02)*	*NC*
*X2B*	0.952 ± 0.038	0.005 ± 0.001	0.974 ± 0.038	0.505 ± 0.003
*X2B2*	0.964 ± 0.020	0.004 ± 0.001	0.984 ± 0.017	0.505 ± 0.003

**Table 6:** Comparison of X2B and X2B2 in terms of maze, maxe, and maye metrics in mm

Method	*maxe*	*maye*	*maze*
*X2B*	2.821 ± 2.717	2.515 ± 2.320	2.590 ± 2.443
*X2B2*	2.473 ± 2.143	2.431 ± 2.212	2.441 ± 2.247

Table 6 highlights X2B2’s improved reconstruction accuracy over X2B in maxe, maye, and maze, showing lower mean errors across all axes. These results reflect X2B2’s ability to reduce positional errors through bi-planar input processing and multi-view data fusion.

**Fig. 7:**
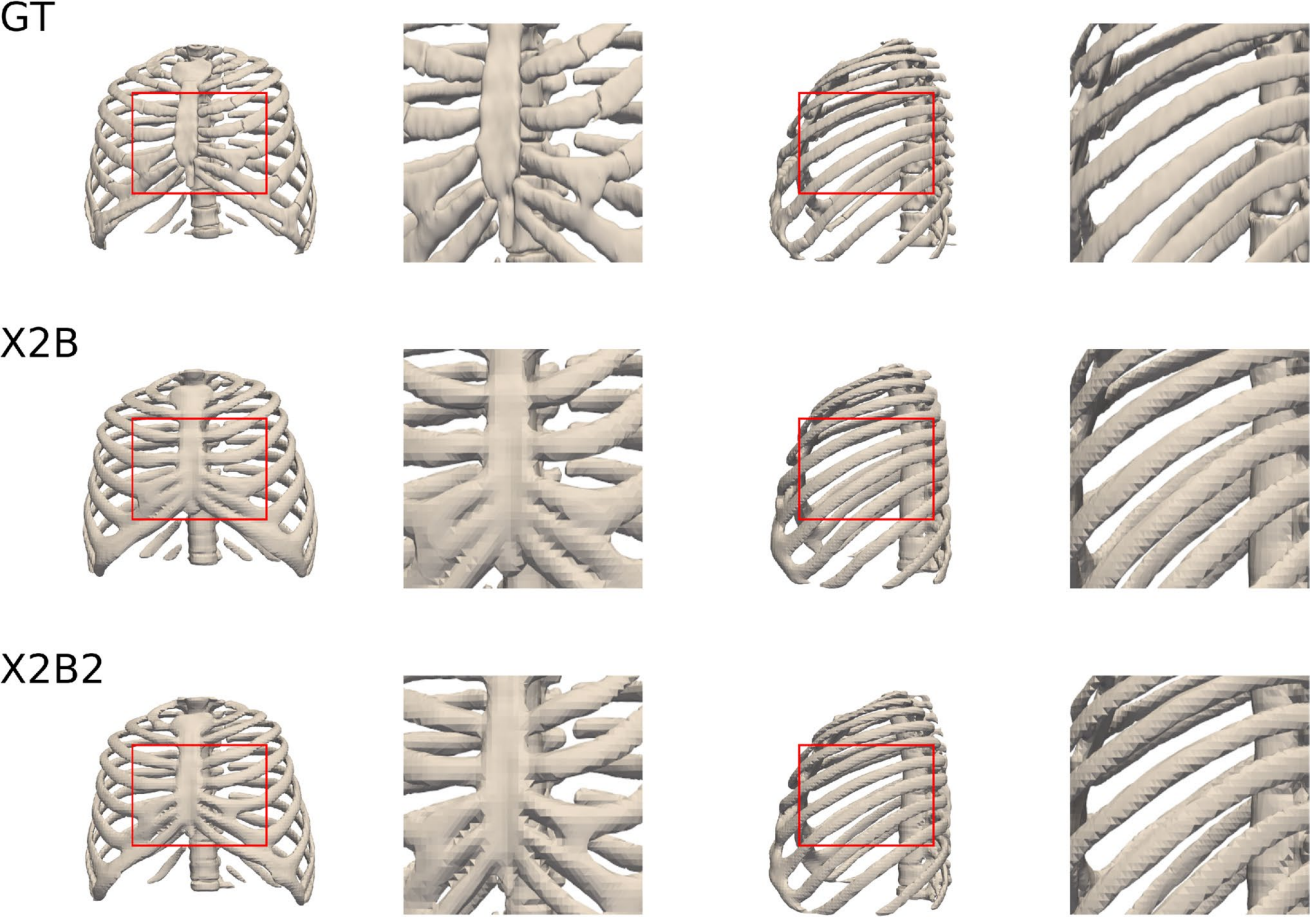
Thorax reconstruction results for GT, X2B, and X2B2. Each row shows full thorax views (front and side) and corresponding zoom-ins to highlight anatomical details

Figure 7 compares thorax reconstructions for the GT, X2B, and X2B2 models. The zoomed-in regions emphasize X2B2’s advantage in capturing finer anatomical details, illustrating its incremental improvement over X2B in reconstructing complex thorax structures.

X2B2 shows a slight numerical advantage over X2B, particularly in preserving fine anatomical details in 3D reconstructions. By leveraging bi-planar DRR inputs and cross-attention, it enhances spatial feature fusion, improving reconstruction accuracy. These refinements make X2B2 well-suited for multi-perspective 3D reconstruction while maintaining X2B’s efficiency. However, since bi‑planar acquisitions are less common in routine thoracic imaging and entail additional radiation and setup, X2B2 is presented only as an ablation study rather than a primary deployment model, whereas our main clinical focus is on the single‑view X2B/X2BR pipeline.

## Conclusion

This study presents X2B and X2BR, two complementary neural implicit frameworks for high-fidelity 3D skeletal reconstruction from a single planar X-ray. X2B leverages a ConvNeXt-based encoder and Conditional Batch Normalization (CBN) layers to predict continuous occupancy fields, enabling template-free reconstruction of complex anatomical structures such as ribs and vertebrae. X2BR builds upon this by integrating a biomechanical template and applying non-rigid registration through GBCPD++, enhancing anatomical plausibility and improving alignment to patient-specific skeletal morphology.

Both frameworks capitalize on the strengths of neural implicit representations—modeling continuous volumetric structures without reliance on voxel grids—while addressing key challenges in X-ray-based reconstruction, including occlusion, overlapping intensities, and incomplete input data. Evaluations on clinical datasets demonstrate that X2B achieves state-of-the-art accuracy in metrics such as volumetric IoU, Chamfer-L1 distance, and F-score, while X2BR offers improved anatomical consistency through template-guided refinement.

For scenarios in which quantitative accuracy and computational throughput are the dominant requirements—such as large-scale cohort analysis or automated severity scoring—we recommend the template-free implicit model X2B. It delivers the best global metrics while completing inference in $\approx 0.24$ s per volume on an RTX-4090, and X2BR likewise operates in the sub-second range with only a modest additional cost for the GBCPD++ registration step. Conversely, when the downstream task demands anatomical fidelity, e.g. surgical planning, patient-specific finite-element simulation, or clinician–patient visualization, the template-refined variant X2BR is preferred.

In addition to methodological advances, this work introduces the largest known dataset of paired 3D bone meshes and corresponding digitally reconstructed radiographs (DRRs), contributing a valuable benchmark for future research. By addressing long-standing limitations in 3D reconstruction from sparse imaging data, X2B and X2BR offer practical tools for surgical planning, orthopedic assessment, and personalized biomechanical simulations.
